# Dental Teaching

**Published:** 1872-08

**Authors:** W. D. Tremper


### Dental Teaching,

BY DR. W. D. TREMPER.

*Read before the Michigan State Dental Society.*

This paper might properly be headed with the interrogative "For What Purpose are Dental Schools Established?" They are intended to advance the standard of professional skill, both operative and mechanical. But are the Dental Colleges of our land accomplishing that work for which they are intended? I think that this is a question which demands the attention of the profession at the present time. We, as dentists, feel that we deserve some protection from the charlatans, who cover their evil practices and impositions with the bombastic title of Surgeon Dentist, &c. The men who are true to our noble profession have long sought this protection by laws enacted by the Legislatures of the several States. In a few instances they have been successful in procuring the passage of laws to regulate the practice of dentistry. But even where these are in force they seem to be only a partial success, so far as aiding the people in distinguishing the true professional dentist from the quack is concerned. The only resource then, seems to be our colleges. We have considered a diploma, granted by one of those institutious, a safeguard and protection to the individual who has faithfully pursued its required course of study, and honorably acquitted himself of the duties therein devolved, from the horde of quacks who crowd the profession. It should be regarded as a recommendation from the faculty of the college from which we graduate. For my part I so considered mine, and it was one of the proudest moments of my life when I received it. The fact that it requires so great an effort, and the proper qualification on the part of the candidate for graduation is what gives this diploma so much value in his eyes, and from this fact the people may be taught to put their confience in the professional skill of the possessor of one. It is only by the Dental Colleges keeping their standard of qualification to the proper point, and thus giving the certificate or diploma which they award, the value which it should have in the estimation of the profession and people, that they can accomplish the work which is expected of them. If it should be known by the people that there existed an easier method by which these diplomas might be obtained, what effect would it probably have on young men about to enter the profession ? What inducement is there to a young man who contemplates the study of dentistry, to take the legitimate way by hard study and attendance of two years at college to get a diploma, when it is known that another, for a stipulated pecuniary consideration, and passing a cursory examination (or none at all), can obtain one in every way just as good in the eyes of the public ? I leave the question for you who are met here to promote the best interests of our chosen profession, to answer. Some of you may think I talk at random, but I assure you I do not. Some of our oldest and highly esteemed dental schools are doing this persistently. Does this have a tendency to advance the standard of professional education? If it does we fail to see it. If they are run as financial machines, designed only to pay as large a dividend as possible to the stockholders, then let it be known—we will not ask of them more than they profess to give. While the professors in these schools are traveling around to Dental Conventions, preaching dental ethics, why don't they teach it to the students under their instruction? When we see graduates from these colleges exhibiting specimens of their work at agricultural fairs, and spreading their commercial advertisements along the roadside fences, we are not so much surprised when we consider what a deficiency there is in this department of dental education. Men are only too prone to regard the profession of dentistry as a mere trade, and it is the duty of those claiming to be dental teachers, to do all in their power to eradicate this idea from their minds. At the present time they are teaching the very contrary—by example, at least. With such an example set before the young men just starting out in our profession how much will our Code of Ethics be worth in their estimation? We reply—less than nothing, for it will only serve to make us appear as hypocrits.

Then let the voice of this Association be heard in favor of keeping the professional standard to the proper elevation. If it be necessary to censure those we have been used to regard as teachers in the profession, let it be done. They surely can accomplish little good while pursuing their present course.

